# Supplementary material for: Distinct subcellular autophagy impairments in induced neurons from patients with Huntington's disease
Source: Brain. 2021 Dec 22;145(9):3035–57. doi: 10.1093/brain/awab473 (PMC9473361; doi:10.1093/brain/awab473)
Supplement: awab473_Supplementary_Data [file awab473_supplementary_data.zip › brain-2021-00831-File012.pdf]

Supplementary Table 1 CRISPRi gRNA sequences.

gRNA	Sequence	"Cut"-site	Orientation
Guide 1	CGTCCATCTTGGACCCGTCC	-2	Antisense
Guide 2	AGGTAAAAGCAGAACCTGAG	25	Antisense
Guide 3	CAGAGCCCCATTCATTGCCC	65	Sense
Control guide	TGCGAATACGCCCACGCGAT	N/A	N/A

Supplementary Table 2 List of antibodies used for ICC and IHC.

Antibody	Conc.	Species	Company	Cat#	RRID
MAP2	1:500	Rabbit	Millipore	Ab5622	AB_91939
MAP2	1:10,000	Chicken	Abcam	Ab5392	AB_2138153
EEA1	1:1,000	N/A	Abcam	Ab109110	AB_10863524
RAB11	1:200	Rabbit	Abcam	Ab3612	AB_10861613
P62	1:500	Rabbit	Abcam	Ab91526	AB_2050336
LC3B	1:500	Rabbit	Sigma	L7543	AB_796155
LAMP1 (clone H4A3)	1:200	Mouse	DSHB	N/A	AB_2296838
TAU (clone HT7)	1:500	Mouse	Thermo Scientific	MN1000	AB_2314654
TAU	1:1,000	Rabbit	Dako	A0024	AB_10013724
Neurofilament Protein antibody	1:200	Mouse	Agilent	M0762	AB_2314899
Alexa Fluor® 488 AffiniPure Donkey IgG	1:200	Rabbit	Jackson	711-545-152	AB_2313584
Alexa Fluor® 488 AffiniPure Donkey IgG	1:200	Mouse	Jackson	715-545-150	AB_2340846
Cy™3 AffiniPure Donkey IgY	1:200	Chicken	Jackson	703-165-155	AB_2340363
Cy™3 AffiniPure Donkey IgG	1:200	Rabbit	Jackson	711-165-152	AB_2307443
Cy™3 AffiniPure Donkey IgG	1:200	Mouse	Jackson	715-165-151	AB_2315777
Alexa Fluor® 647 AffiniPure Donkey IgG	1:200	Mouse	Jackson	715-605-150	AB_2340862

Supplementary Table 3 List of antibodies used for WB

Antibody	Conc.	Species	Company	Cat#	RRID
BECN1	1:500	Rabbit	Santa Cruz	sc-11427	AB_2064465
P62	1:5,000	Mouse	Abcam	ab56416	AB_945626
LC3B	1:5,000	Rabbit	Sigma	L7543	AB_796155
LAMP1 (clone H4A3)	1:1,000	Mouse	DSHB	N/A	AB_2296838
Hrp Conjugated antibody	1:2,500	Rabbit	Sigma-Aldrich	NA9340	AB_772191
Hrp Conjugated antibody	1:5,000	Mouse	Santa Cruz	Sc-2005	AB_631736
β-actin	1:100,000	Mouse	Sigma-Aldrich	A3854	AB_262011
