# Supplementary material for: Distinct subcellular autophagy impairments in induced neurons from patients with Huntington's disease
Source: Brain. 2021 Dec 22;145(9):3035–57. doi: 10.1093/brain/awab473 (PMC9473361; doi:10.1093/brain/awab473)
Supplement: awab473_Supplementary_Data [file awab473_supplementary_data.zip › brain-2021-00831-File010.pdf]

Supplementary Information

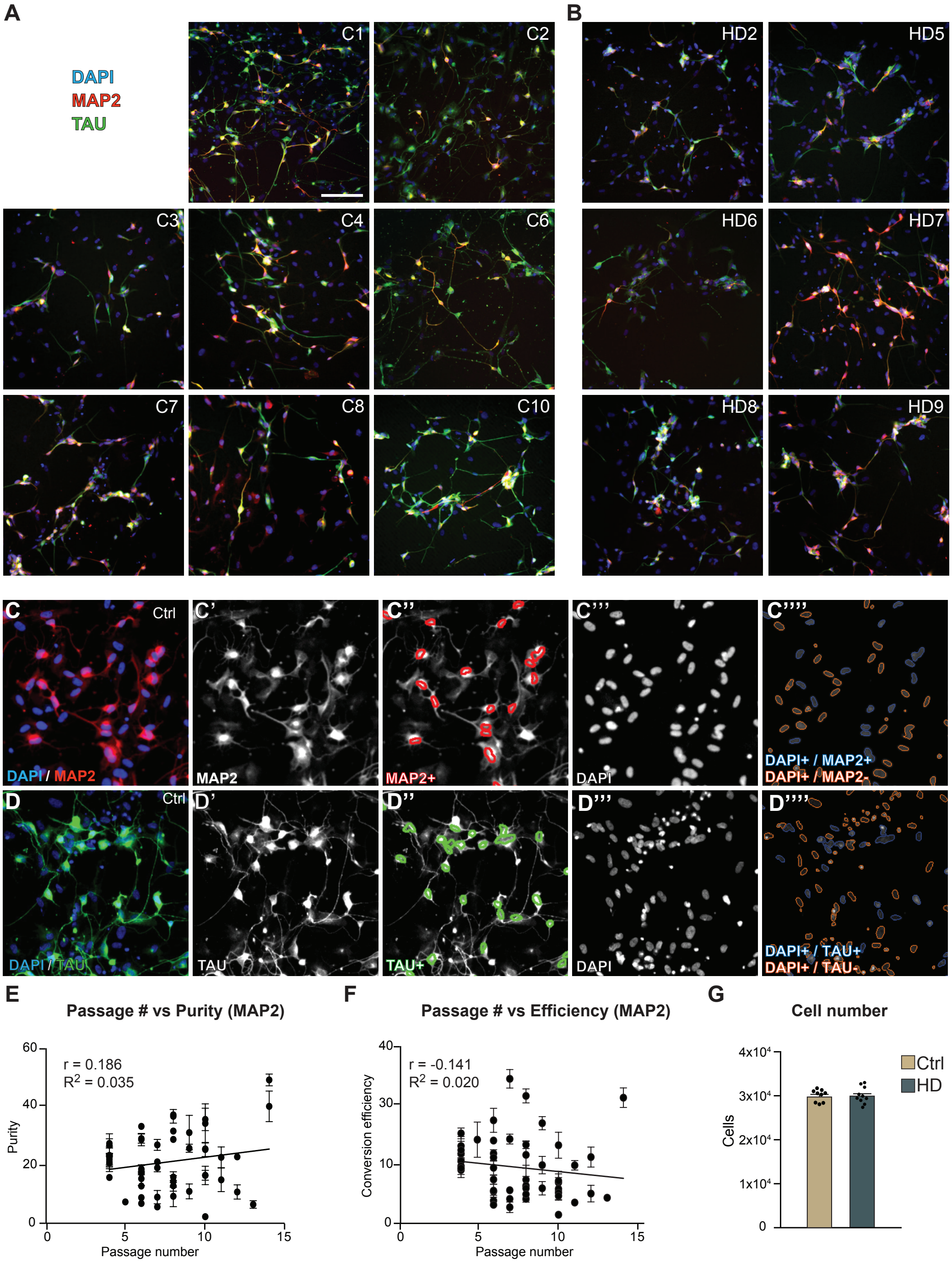

Supplementary Fig. 1. iNs display a neuronal profile. Related to Figure 1.

(**A-B**) Fibroblast-derived iNs from control and HD patients express mature neuronal markers like TAU and MAP2. (**C-D**) Target activation image analysis by high-content automated microscopy. DAPI⁺ cells are defined by intensity and area criteria. Border objects are excluded.

MAP2$^+$ and TAU$^+$ iNs are defined by average and total intensity measurements on a cell-by-cell basis. On c''''and d'''' blue circles around the nuclei define the valid MAP2$^+$ or TAU$^+$ iNs, while orange circled nuclei are not positive for MAP2 or TAU and therefore are not counted as converted neurons. Red circles represent MAP2$^+$ iNs in c'', while TAU$^+$ iNs are marked with green circles in d''. (**E-F**) There is no correlation between purity or conversion efficiency and passage number (n = 48 replicates; 195 wells analyzed in total). (**G**) Average DAPI$^+$ cell numbers analyzed with high-content automated microscopy. Each dot represents the average value for one control or HD cell line (n = 9 lines for controls, 81 wells analyzed in total; n = 10 lines for HD, 85 wells analyzed in total).

(*$p<0.05$; Two-tailed Pearson's correlation coefficients were used in e and f. Two-tailed unpaired T-tests were used in g) All data are shown as mean ± SEM. Scale bar is 50 µm.

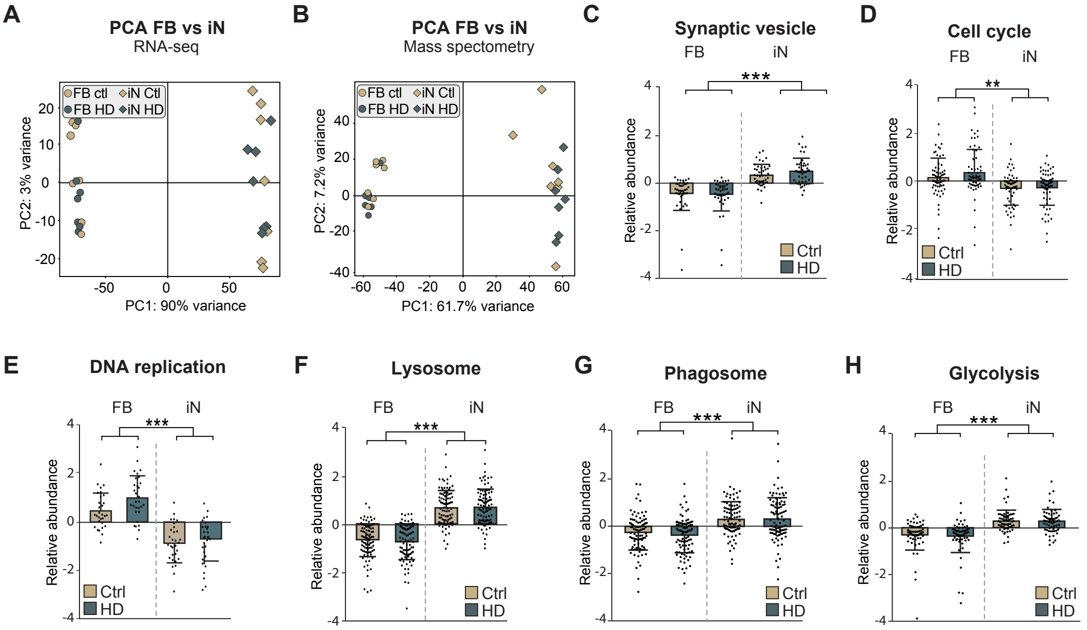

Supplementary Fig. 2. iNs display a neuronal profile at the transcriptome and proteome level. Related to Figure 1.

(**A**) Principal component analysis of transcriptome data of ntop = 500 "top 500 variable genes" showed a major transcriptional difference between fibroblasts and iNs (n = 7 control and 7 HD fibroblast and iN lines). (**B**) Principal component analysis using the protein abundance profiles of all samples under study. The highest variability explained by the first component separates iNs from fibroblasts. There is more variability among iNs than among fibroblasts (n = 7 control and 7 HD fibroblasts and iN lines). (**C-H**) Pathways dysregulated between fibroblasts and iNs. Relative abundance profiles of proteins from several pathways (Cell cycle, DNA replication, Glycolysis, Lysosome, Phagosome and Synaptic vesicle) in the four group of samples (n = 7 control and 7 HD fibroblasts and iN lines).

(***p<0.001; **<0.01; two-tailed paired T-tests were used in c-h. Data shown as mean ± SD)

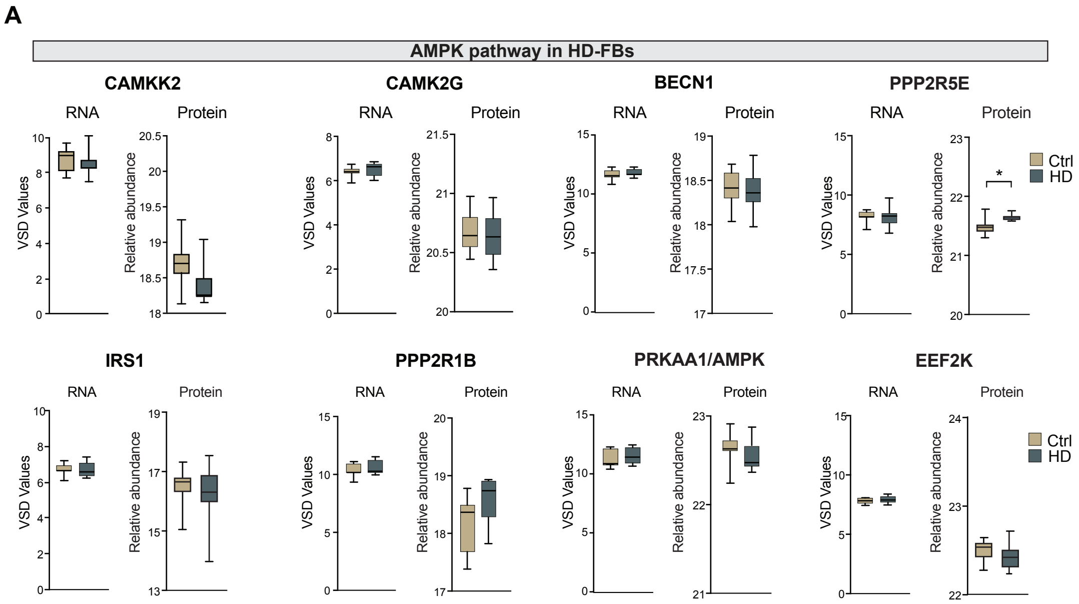

Supplementary Fig. 3. Related to Figure 2.

(**A**) Protein abundance and RNA expression of CAMKK2, CAMK2G, AMPK, PPP2R1B, PPP2R5E, IRS1, EEF2K and BECN1 in control and HD-FBs (n = 7 control and 7 HD fibroblast lines).

(*p<0.05; two-tailed unpaired T-tests were used) All data are shown as min/max box plots.

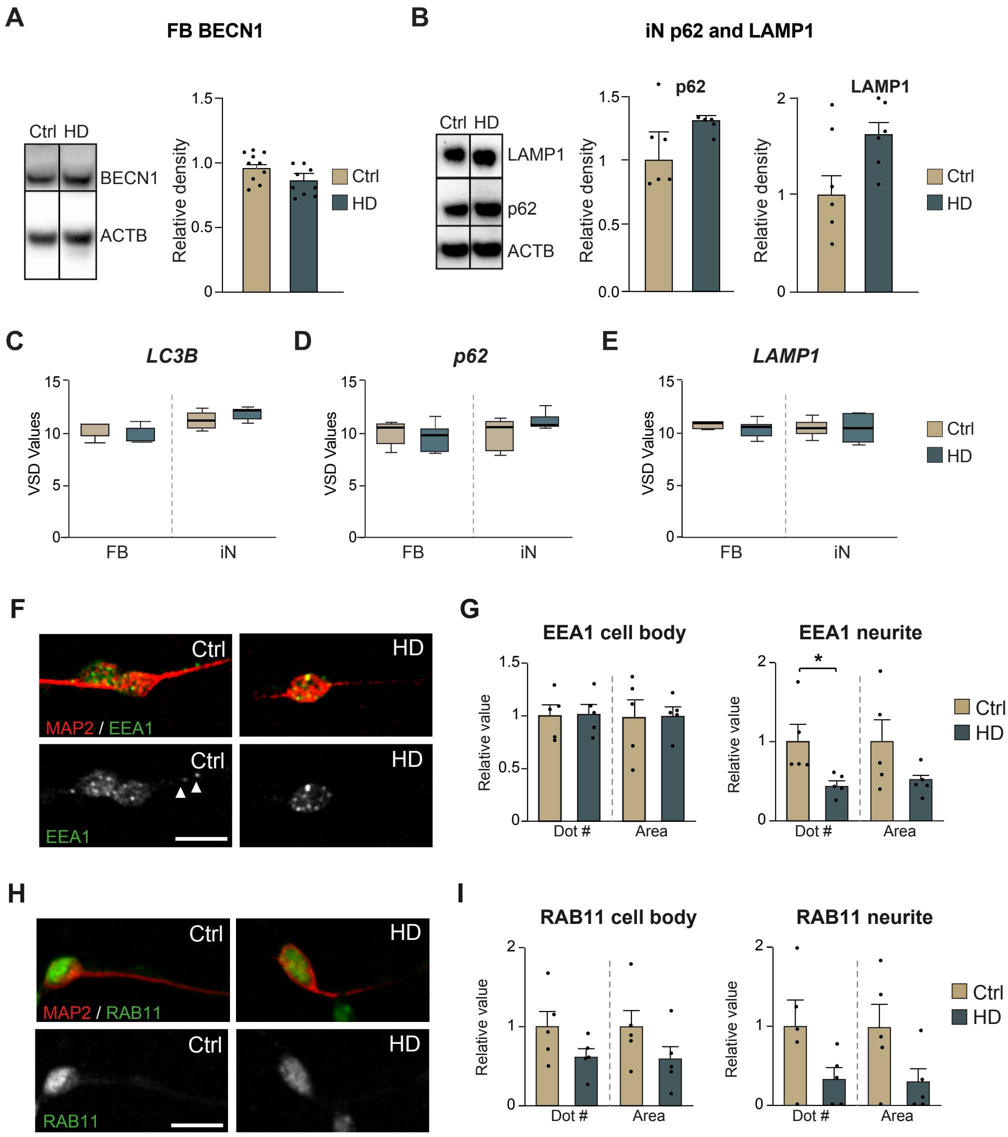

Supplementary Fig. 4. Related to Figure 3.

(**A**) BECN1 expression was not changed in ctrl and HD fibroblasts using WB (n = 10 replicates). (**B**) p62 and LAMP1 expression was similar between the control and HD-iNs (n = 6 replicates). (**C-E**) VSD values of *LC3B*, *p62* and *LAMP1* from RNA sequencing of control and HD fibroblasts and iNs (n = 7 control and 7 HD fibroblasts and iN lines). (**F-I**) Representative images and statistical analysis of EEA1 and RAB11 dot number and size in MAP2+ cell bodies and neurites of HD-iNs compared to controls (n = 5 lines).

(*$p<0.05$; two-tailed unpaired T-tests were used). All data are shown as mean ± SEM in a, b, g and i. All data are shown as min/max box plots in c. WB values were normalized to non-

treated control fibroblasts or iN expression levels and corrected to actin values. Scale bar is 25 μm.

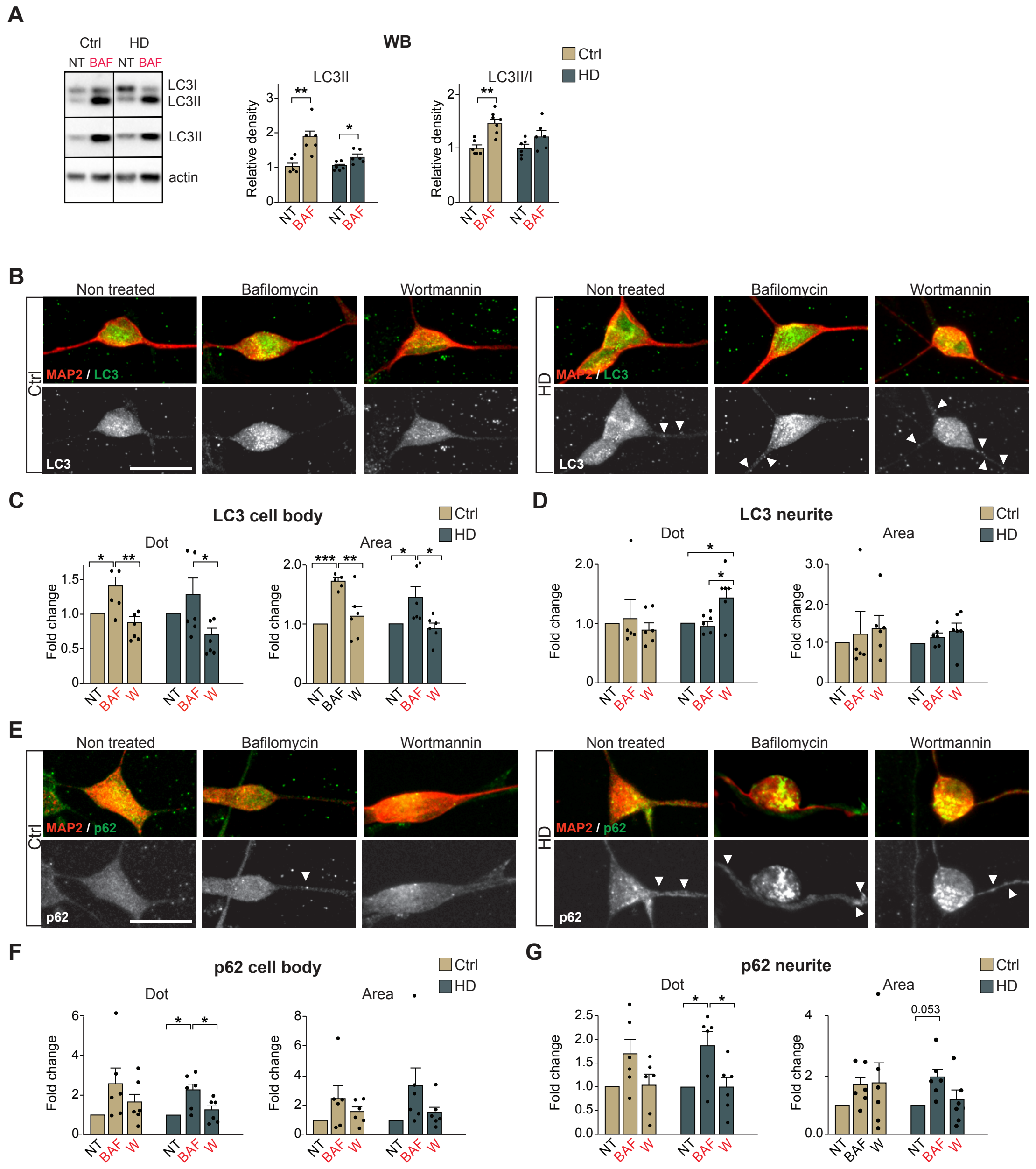

Supplementary Fig. 5. Related to Figure 4.

(**A**) LC3B-II levels are significantly increased both in the HD-iNs and ctrl-iNs after BAF treatment, while the LC3B-II/LC3B-I ratio only increased in the healthy ctrl-iNs but not in the HD-iNs after the treatment (n = 6 replicates). (**B-D**) Representative images and fold changes of LC3B spot count and area in the cell body and in the neurites in control and HD-iNs after Bafilomycin A1 (Baf) and Wortmannin (W) treatment. Non-treated (-) samples were set to 1 (n = 6 ctrl and HD-iN lines). (**E-F**) Representative images and fold changes of p62 spot count

and area in control and HD-iN cell bodies and neurites after BAF and W treatment (n = 6 ctrl and HD-iN lines).

(***$p<0.001$; **<0.01; *$p<0.05$; One-way ANOVA or nonparametric Kruskal-Wallis test was used depending on normal distribution defined by D'Agostino-Pearson omnibus normality test for b-c and e-f. Two-tailed paired T-tests were used in panel a). All data are shown as mean ± SEM. WB values were normalized to ctrl- iNs expression levels and corrected to actin values. Scale bar is 25 μm. FC: Fold-change; NT: Non-treated; BAF or B: Bafilomycin A1; W: Wortmannin

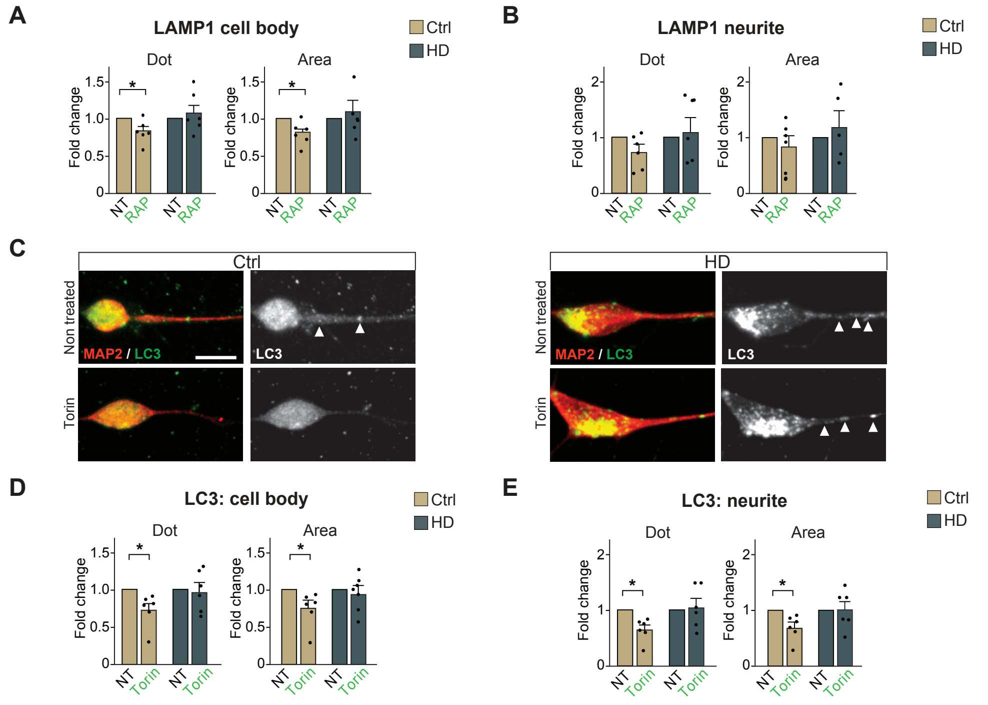

Supplementary Fig. 6. Related to Figure 4.

(**A-B**) Statistical analysis after rapamycin treatment of LAMP1 spot number and size in control and HD-iNs. Non-treated (-) samples were set to 1 (n = 6 ctrl and HD-iN lines). (**C-E**) Representative images and fold changes of LC3B spot count and area in control and HD-iN cell bodies and neurites after torin treatment (n = 6 ctrl and HD-iN lines).

(*$p<0.05$; Two-tailed paired T-tests were used). All data are shown as mean ± SEM. Scale bar is 25 µm. NT: Non-treated; RAP: Rapamycin

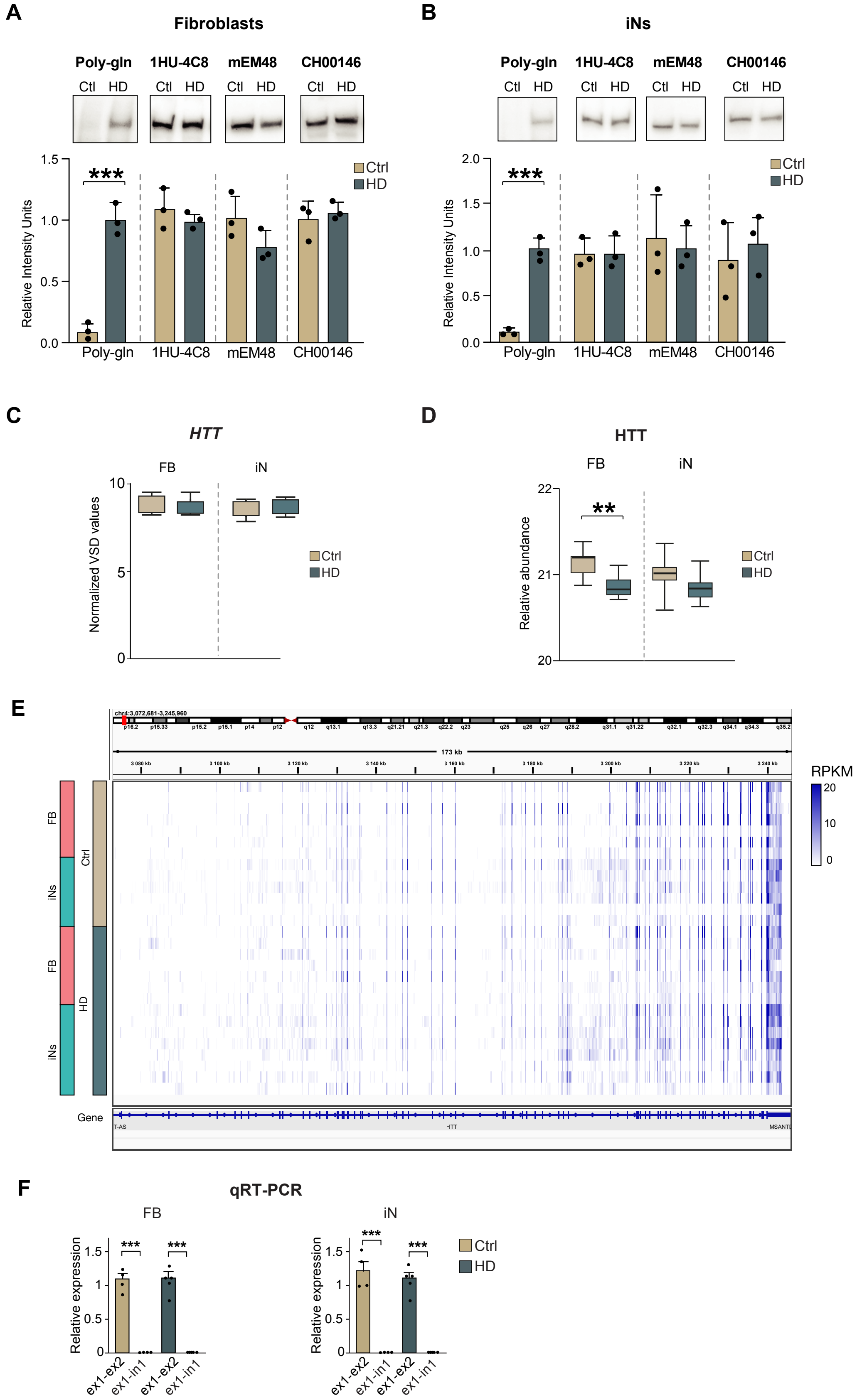

A
Fibroblasts
Poly-gln
1HU-4C8
mEM48
CH00146
Ctl HD
Relative Intensity Units
***
Ctrl
HD
B
iNs
C
HTT
FB
iN
Normalized VSD values
D
HTT
Relative abundance
**
E
RPKM
FB
iNs
Ctrl
HD
Gene
HTT
F
qRT-PCR
Relative expression
ex1-ex2
ex1-in1

Supplementary Fig. 7. Expression of HTT protein in fibroblasts and iNs without protein aggregation.

(**A-B**) Western blot quantification and representative immunoblots of HTT levels in fibroblasts (n = 3) and iNs (n = 3) using Poly-gln, 1HU-4C8, mEM48 and CH00146 antibodies. (**C**) *HTT* RNA normalized VSD counts from control and HD fibroblasts and iNs after RNA-sequencing (n = 7 control and 7 HD fibroblast and iN lines). (**D**) Protein abundance of HTT in control and HD fibroblasts and iNs using MS (n = 7 control and 7 HD fibroblasts and iN lines). (**E**) IGV tracks as heatmap, showing RPKM values over *HTT*. Row annotation showing if a sample is fibroblast or iNs, ctrl or with HD. (**F**) Using 3 housekeeping genes (HPRT1, GAPDH and ACTIN) we could not detect any expression by using exon 1 - intron 1 primers in the fibroblasts or the iNs, while there was a clear expression of *HTT* using exon 1 - exon 2 primers (n = 4 lines for ctrl and n = 5 lines for HD).

(p***<0.001; p**<0.01; two-tailed unpaired T-tests were used) All data are shown as mean ± SD. All data are shown as min/max box plots in b and c. WB values are normalized relative intensity units corrected to total protein values.

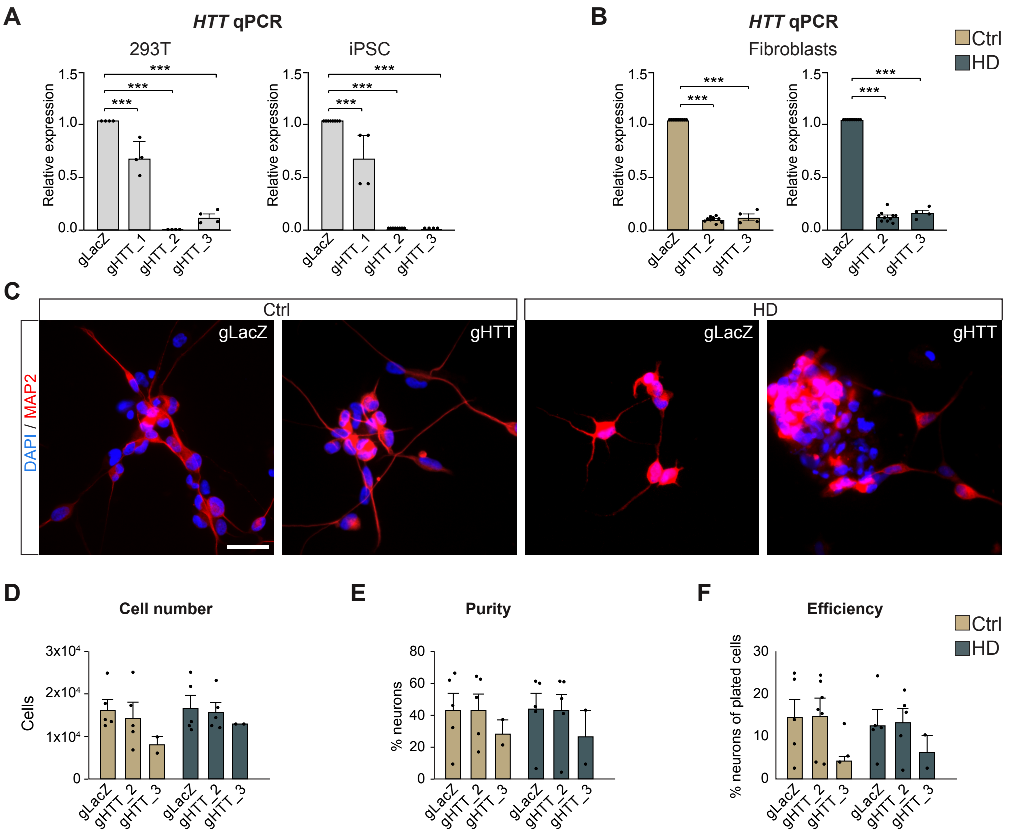

Supplementary Fig. 8. Efficient silencing of *HTT* using CRISPRi. Related to Figure 5.

(**A**) 293T and iPS qRT-PCR representing silencing efficiency of the *HTT* gene using three gRNAs and vectors with MOI 10 (n = 4 for 293T cells and n = 4 for iPSc). (**B**) qRT-PCR representing silencing efficiency of the *HTT* gene in control and HD fibroblasts using g2RNA and g3RNA (n = 10 for LacZ and gRNA2 from 5 ctrl and 5 HD-iN lines and n = 4 for gRNA3 from 2 ctrl and 2 HD-iN lines). (**C**) Representative images of *HTT* and *LacZ* silencing $MAP2^+$ control and HD-iN cells using CRISPRi. (**D**) Average $DAPI^+$ cell numbers analyzed with high-content automated microscopy in *LacZ* and *HTT* g2RNA and g3RNA transduced control and HD-iNs (n = 5 ctrl and HD-iN lines for LacZ and gRNA2, n = 2 ctrl and HD-iN lines for gRNA3). (**E-F**) Purity and conversion efficiency of *HTT* and *LacZ* g2RNA and g3RNA transduced control and HD-iNs (n = 5 ctrl and HD-iN lines for LacZ and gRNA2, n = 2 ctrl and HD-iN lines for gRNA3).

(***p<0.001; **<0.01; *p<0.05; Ordinary one-way ANOVA was used in a and b. Two-way ANOVA was used in d-f.) All data are shown as mean ± SEM. Scale bar is 50 μm.

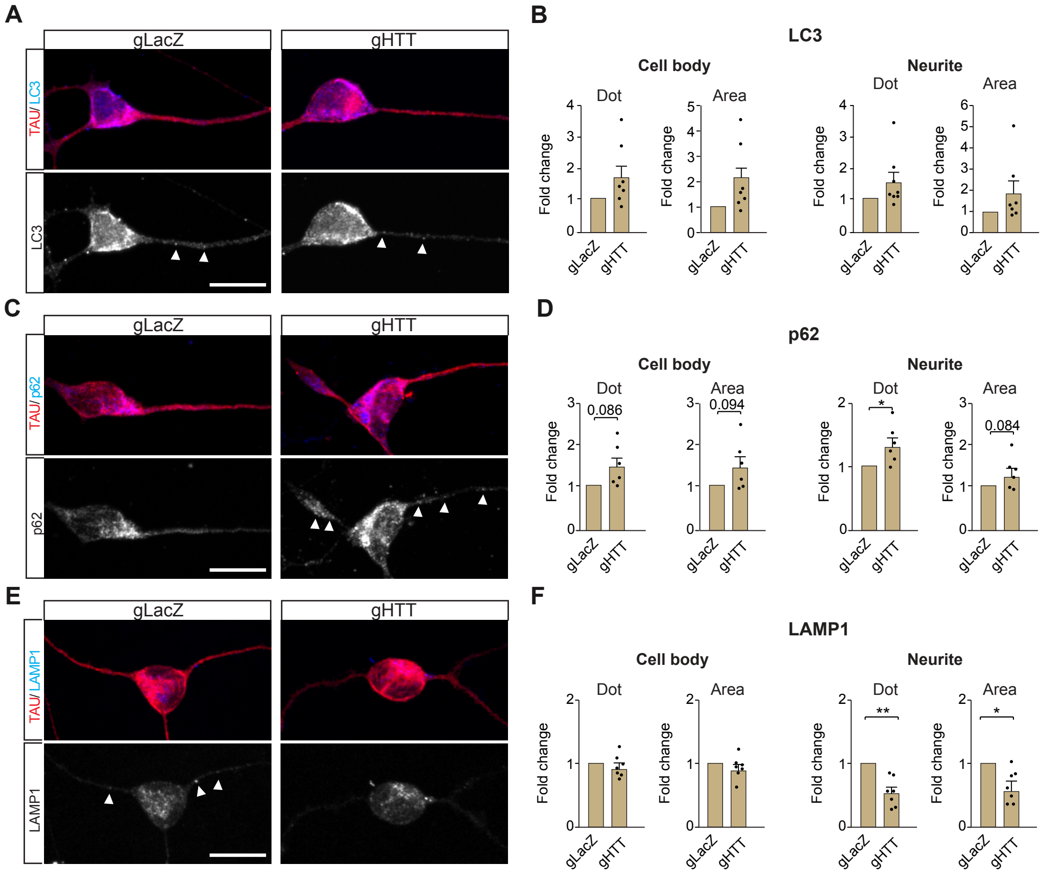

Supplementary Fig. 9. Related to Figure 5.

(**A-F**) Representative images and statistical analysis of LC3B, p62 and LAMP1 dot number and area in TAU⁺ cells in control iNs stably expressing LacZ and HTT gRNAs using CRISPRi (n = 7 replicates from 5 ctrl and 5 HD-iN lines pooling gRNA2 and gRNA3 data). (*p<0.05; Two-tailed paired t-tests were used.) All data are shown as mean ± SEM. Fold changes are presented in all graphs. Scale bar is 25 µm.

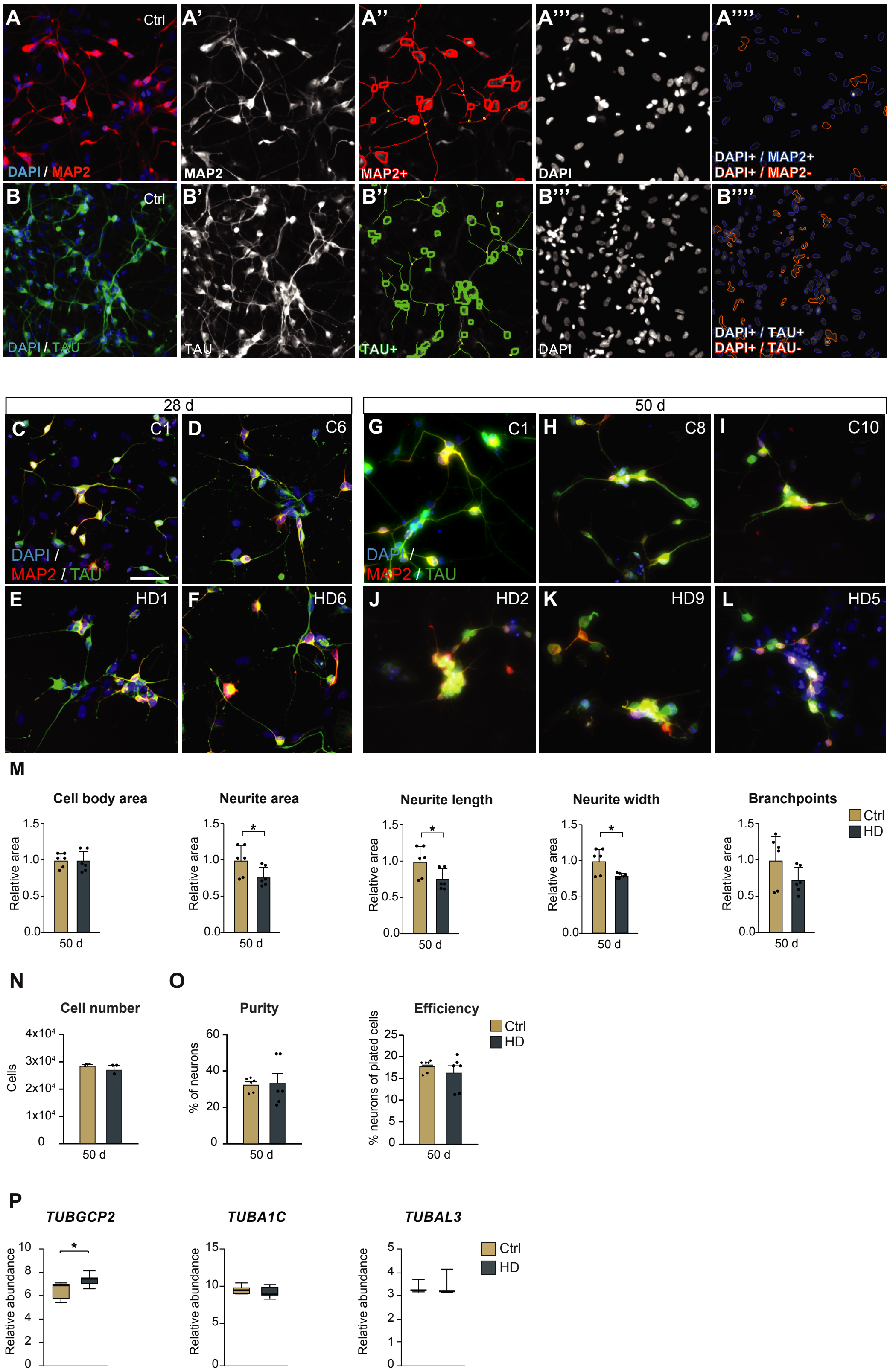

Supplementary Fig. 10. Related to Figure 6.

(**A-B**) Neuronal profiling image analysis by high-content automated microscopy. DAPI$^+$ cells are defined by intensity and area criteria. Border objects are excluded. MAP2$^+$ or TAU$^+$ cell bodies are defined by intensity measurements and area and shape on a cell-by-cell basis. Blue circles around the nuclei define the valid MAP2$^+$ or TAU$^+$ iNs, while orange circled nuclei are not positive for MAP2 or TAU and therefore are not counted as converted neurons. Red circles and neurites represent MAP2$^+$ iNs while green circles and neurites represent TAU$^+$ iNs. (**C-L**) Representative ICC images from control and HD patient iNs after 28 and 50 days of conversion. (**M**) Neuronal morphology measurement comparing control and HD-iNs after 50 days of conversion (n = 6 replicates from 3 ctrl and 3 HD-iN lines) (**N**) Average DAPI+ cell numbers analyzed with high-content automated microscopy after 50 days of neuronal conversion. Each dot represents the average value for one control or HD cell line (n = 3 lines). (**O**) Percentage of MAP2+ and TAU+ neurons from DAPI+ cells. Each dot represents the average value for one control or HD cell line. Percentage of MAP2+ and TAU+ neurons from plated cells after conversion (n = 6 for controls and HD). (**P**) RNA expression of three different tubulin genes in control and HD-iNs (n = 7 ctrl and 7 HD-iN lines).

(*$p<0.05$; two-tailed unpaired T-tests were used) All data are shown as mean ± SEM in m-o. All data are shown as min/max box plots in p. Scale bar is 50 μm.
