# Supplementary material for: Distinct subcellular autophagy impairments in induced neurons from patients with Huntington's disease
Source: Brain. 2021 Dec 22;145(9):3035–57. doi: 10.1093/brain/awab473 (PMC9473361; doi:10.1093/brain/awab473)
Supplement: awab473_Supplementary_Data [file awab473_supplementary_data.zip › brain-2021-00831-File011.pdf]

Uncropped blots related to Figure 3a

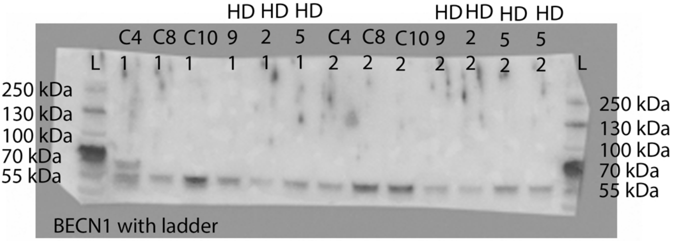

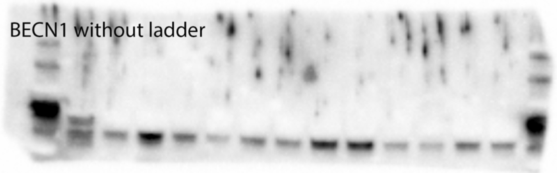

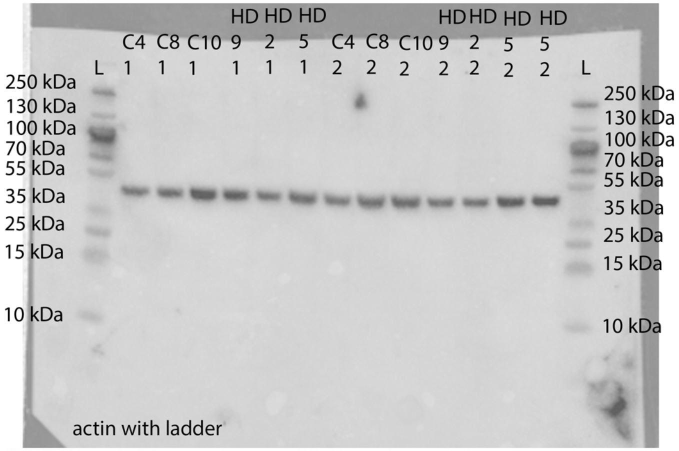

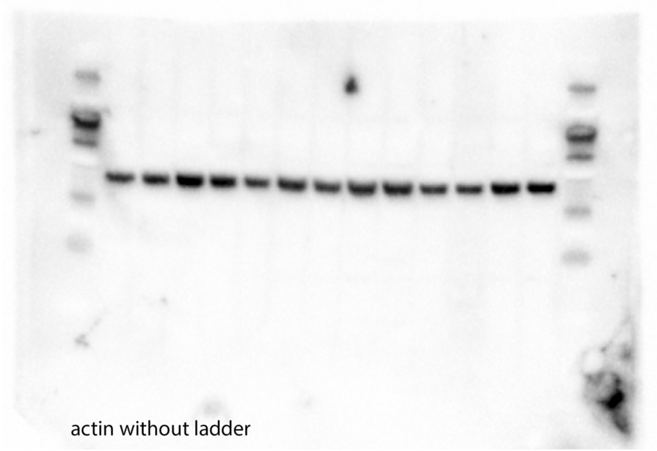

Uncropped blots related to Figure 3b

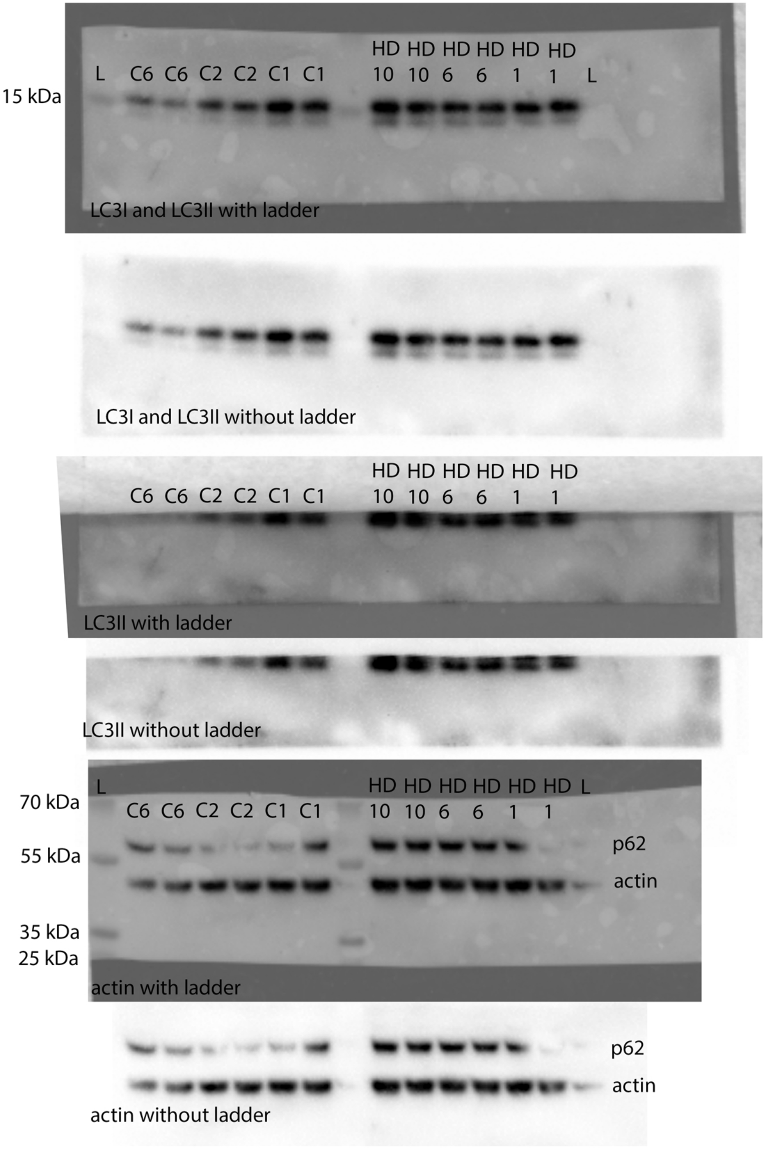

Uncropped blots related to Supplementary Figure 4a

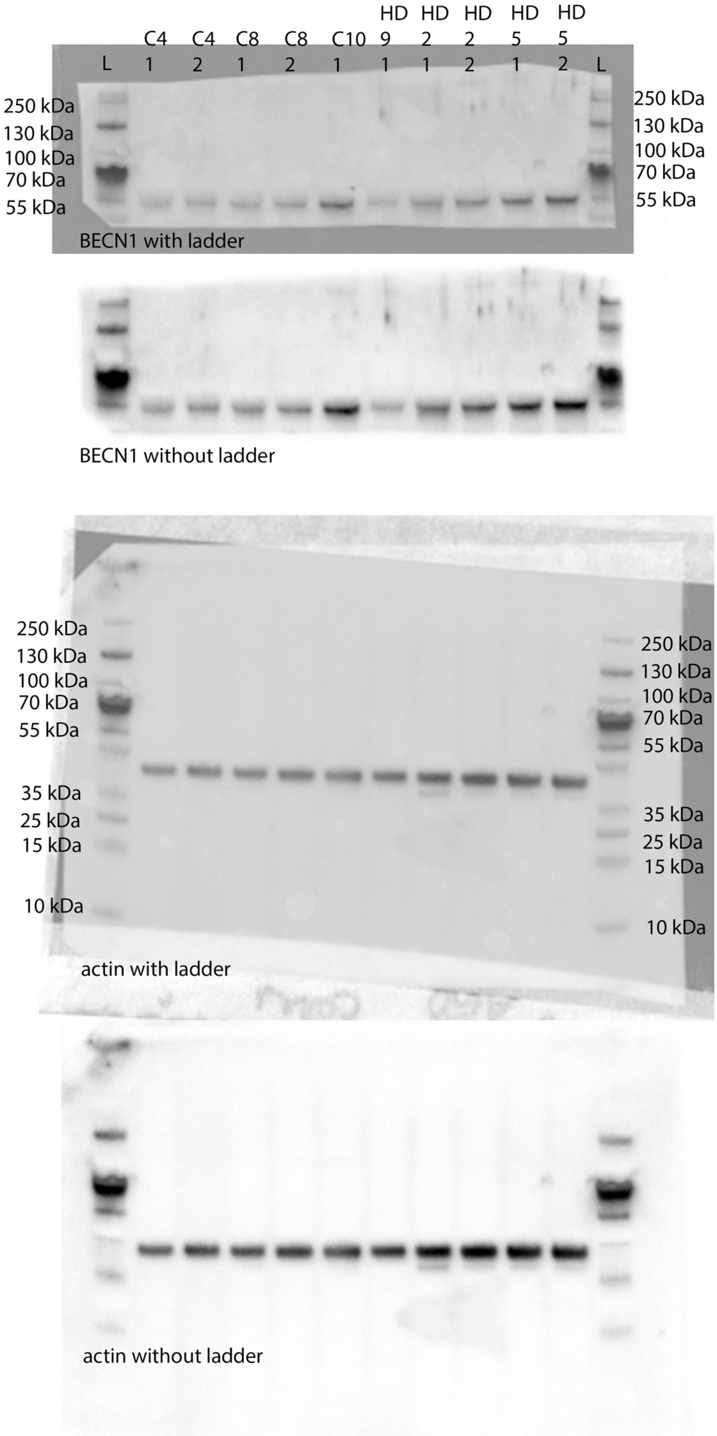

Uncropped blots related to Supplementary Figure 4b

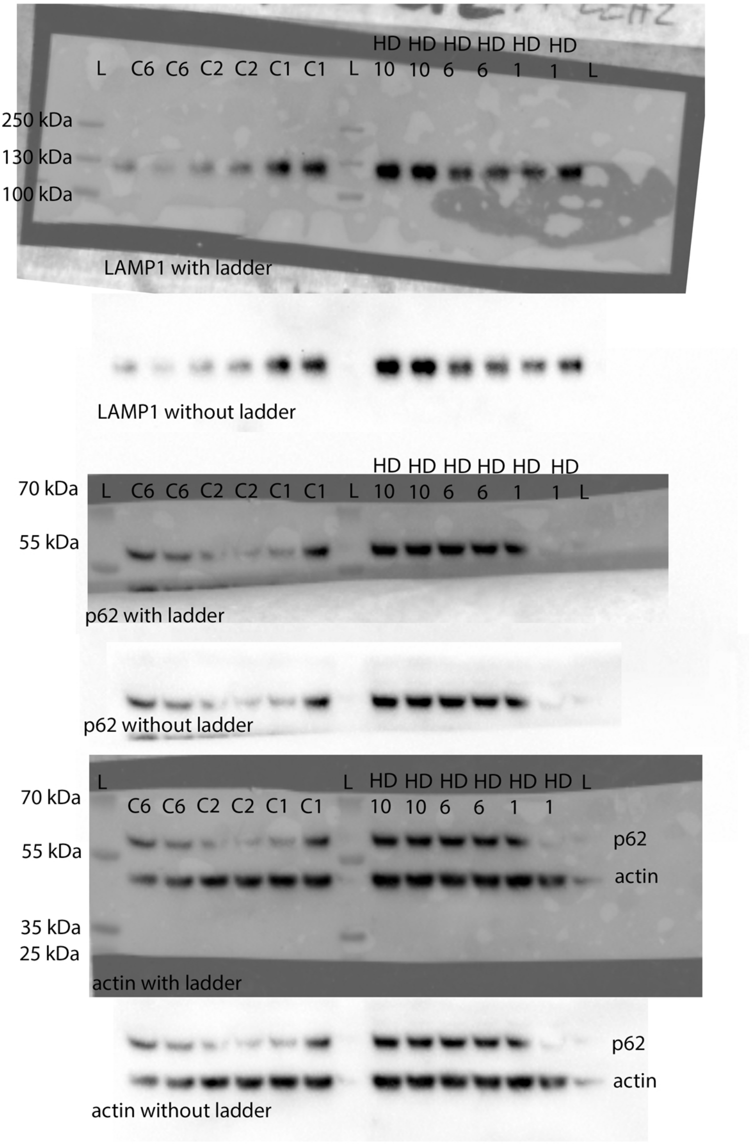

Uncropped blots related to Supplementary Figure 5a

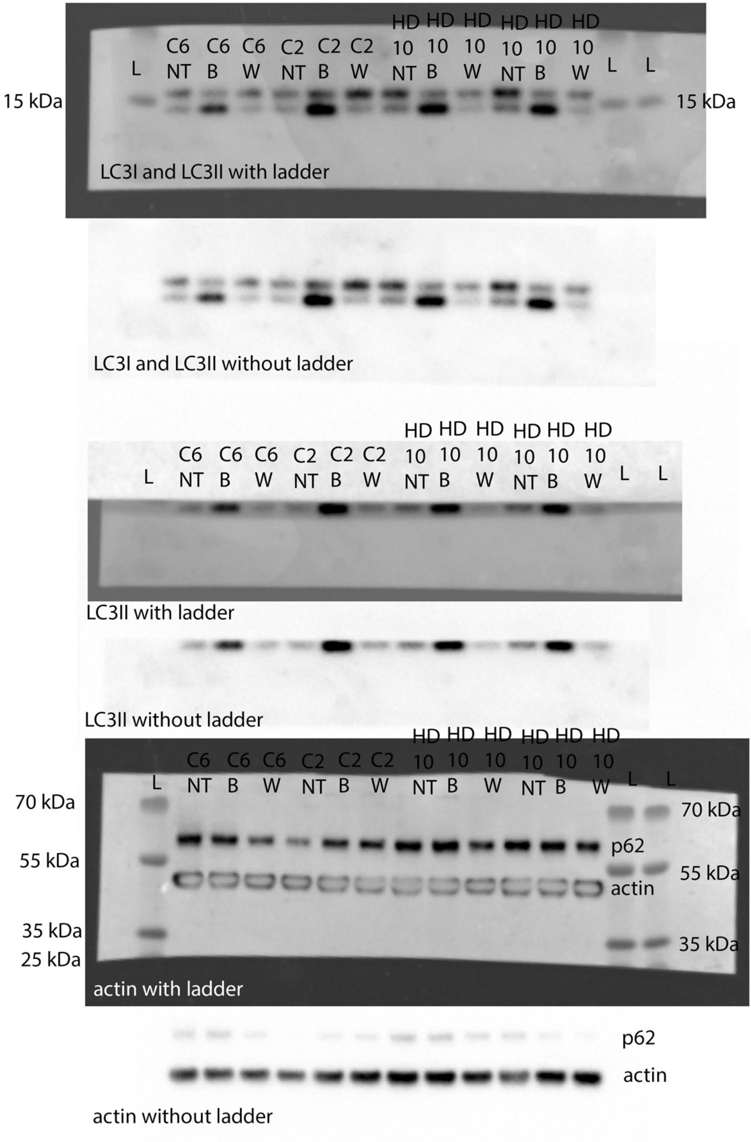

Uncropped blots related to Supplementary Figure 7a and b.

1HU-4C8

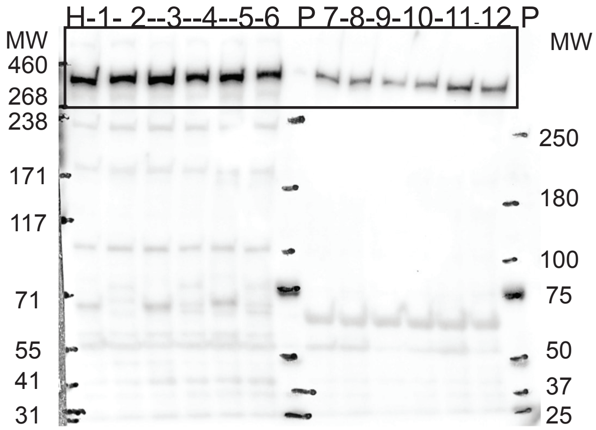

mEM48

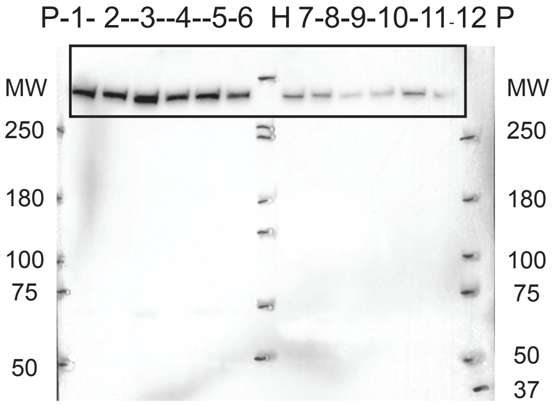

CH00146

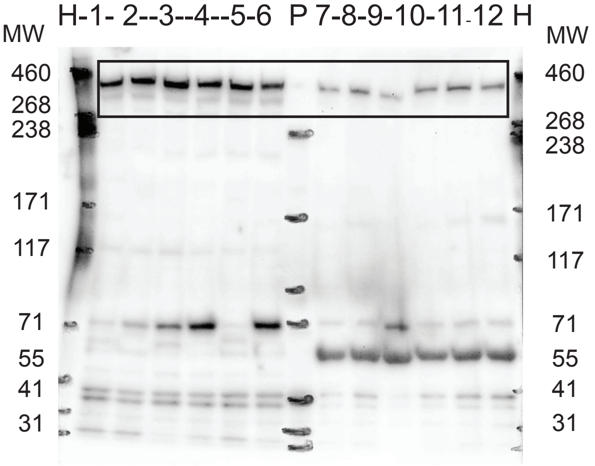

Actin

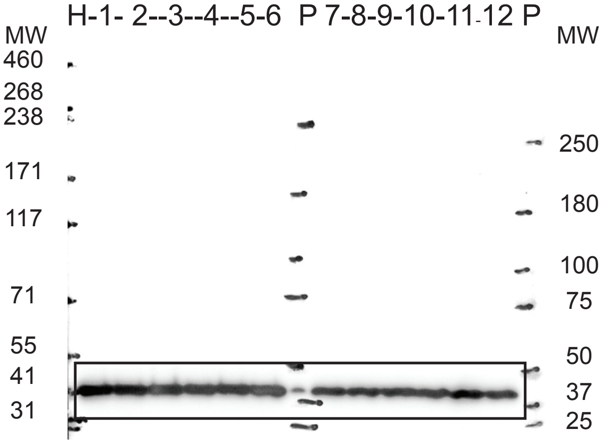

Poly-Gln

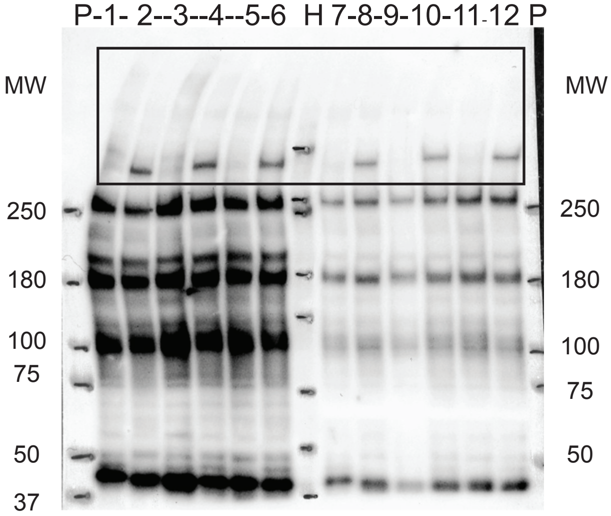

H- High mark MW marker
P- Precision Plus MW marker
1- Fibroblast C1
2- Fibroblast HD1
3- Fibroblast C2
4- Fibroblast HD2
5- Fibroblast C3
6- Fibroblast HD3
7- iN C1
8- iN HD1
9- iN C2
10- iN HD2
11- iN C3
12- iN HD3
